# Improving Population Health Measurement in National Household Surveys: A Simulation Study of the Sample Design of the Comprehensive Survey of Living Conditions of the People on Health and Welfare in Japan

**DOI:** 10.2188/jea.JE20100102

**Published:** 2011-09-05

**Authors:** Nayu Ikeda, Kenji Shibuya, Hideki Hashimoto

**Affiliations:** 1 Department of Global Health Policy, Graduate School of Medicine, The University of Tokyo, Tokyo, Japan; 2 Department of Health Economics and Epidemiology Research, School of Public Health, The University of Tokyo, Tokyo, Japan

**Keywords:** Comprehensive Survey of Living Conditions of the People on Health and Welfare, sample design, simulation, root mean squared error, Japan

## Abstract

**Background:**

The Comprehensive Survey of Living Conditions of the People on Health and Welfare (CSLC) is a major source of health data in Japan. The CSLC is not strictly based on probabilistic sampling, but instead uses an equal allocation of sample clusters to yield equal standard errors of estimates across prefectures. This study compared the performance of this sample design in measuring population health with that of an alternative probabilistic sampling approach.

**Methods:**

A simulation analysis was conducted using hypothetical population data (*n* = 34 262 865) from which 1000 sample datasets were randomly drawn using 2 sampling methods, namely, a conventional stratified random sampling of a constant number of clusters and an alternative 2-stage cluster sampling of households with probability proportional to size. The root mean squared error was used to measure the accuracy of estimated means of a continuous variable and proportions of its dichotomized variable.

**Results:**

The alternative method reduced the variability of estimates in the total population and by strata. It improved further with an increased number of sample clusters in conjunction with a reduced sampling rate of households from selected clusters.

**Conclusions:**

The alternative sample design increased the overall accuracy of population estimates of continuous and dichotomous variables from the CSLC. These benefits should be carefully weighed against the costs incurred in traveling to additional clusters in large prefectures. Further simulation research is necessary to investigate the performance of sampling designs for nominal and ordinal response variables.

## INTRODUCTION

The Comprehensive Survey of Living Conditions of the People on Health and Welfare (CSLC) is a major source of data for tracking trends in population health and for the evaluation of health programs in Japan. The CSLC is a large-scale survey that is conducted every 3 years to provide information for the assessment of health outcomes at the subnational level of 47 prefectures, while small-scale surveys on the status of households and their income are implemented during the interim. In this large-scale survey, to ensure a sufficient sample size and equal errors of estimates across prefectures, a constant number of clusters are randomly selected from prefectures and designated cities with a population of more than 500 000.^1^ For example, 100 clusters are sampled from each prefectures that does not have a designated city, so that target precision for total estimates of households remains approximately 2% to 3% across prefectures.^2^ The clusters are census enumeration areas consisting of 50 households on average,^3^ and all households in the sample clusters are asked to participate in the survey.^2^

The sample design of the CSLC raises 2 issues. First, under an equal allocation of sample clusters, the sample does not reflect the distribution of the total population because the population size substantially differs across prefectures. Thus, in the absence of appropriate adjustment, estimates of population parameters based on such samples may be subject to considerable sampling errors. Although the survey provides a ratio of a sample size to an estimated Japanese population in each prefecture as sample weights, they are only useful for expanding estimated totals of the number of households or household members from a sample to the subnational level, which is a primary purpose of the CSLC. The second problem of this sample design is that the confidentiality of personal information may be violated during the dissemination of secondary data for scientific research. Given that all households in selected clusters are included in a study sample, the possibility cannot be completely excluded that, without data masking, individuals or households might be identified from variables related to the sample design or any other identifying information in secondary data released for public use.

A potential alternative approach to overcome these limitations in the current CSLC sample design is to use 2-stage cluster sampling of households with probability proportional to size. In theory, this sampling procedure allows a sample to be proportional to the distribution of the whole population and may also improve confidentiality, which would be advantageous for respondents. With appropriate sampling fractions, this alternative strategy might be able to maintain the original target sample size of each prefecture in the CSLC. However, it is not known how well this sampling approach compares with the conventional constant cluster sampling of the CSLC in the estimation of population values. This lack of evidence is partly attributable to the fact that population parameters are usually unknown.

This study compared the statistical performance of the conventional and alternative sample designs by conducting a simulation study based on a hypothetical population. The major advantage of this simulation approach is that the known true values (ie, population means and variances) can be used as a benchmark for the assessment of the statistical performance of the sampling strategies. Previous studies have applied simulation techniques to investigate a number of important issues in medical statistics, epidemiology, and other fields.^4^^–^^7^ We hope that the present analysis will provide a useful example of generating evidence for discussions of the establishment of a health information base through the redesign of national household health surveys in Japan.

## METHODS

### Population data

A dataset of a hypothetical population was created for the simulation analysis. The artificial population was intended to be approximately one fifth the size of the population of Japan. The population data had 10 strata, and the numbers of clusters, households, and individuals were generated by pseudorandom number generators with predetermined initial values and distributions. The number of household members of the *j*th household in the *i*th cluster of the *h*th stratum, *N_hij_*, followed the discrete uniform distribution on the integers between 1 and 6:

$$N_{hij}\in [1,2,3,4,5,6],\;j=1,2,\ldots ,N_{hi}.$$

The number of households in the *i*th cluster of the *h*th stratum, *N_hi_*, was distributed normally with a mean of 50 and a variance of 1:

$$N_{hi}\sim N(50,1),\;i=1,2,\ldots ,N_{h}.$$

The mean and variance of *N_hi_* were specified so that cluster sizes were consistent with the sizes of census enumeration areas. The number of clusters in the *h*th stratum, *N_h_*, followed the discrete uniform distribution on the integers between 4000 and 40 000:

$$N_{h}\in [4000,4001,\ldots ,39999,40000],\;h=1,2,\ldots ,10.$$

The range of *N_h_* corresponded to that of the number of census enumeration areas by prefecture in the 2005 Population Census of Japan, which was the sampling frame of the 2007 CSLC.^2^

A continuous random variable *X* was created as a benchmark for assessing the statistical performance of the sample designs. The idea for this variable originated from systolic blood pressure in millimeters of mercury. A normal distribution was assumed in generating pseudorandom numbers for *X* with different means and variations across strata, clusters, and households. *X* was assigned to the *k*th individual of the *j*th household in the *i*th cluster of the *h*th stratum as

$$X_{hijk}\sim N(\mu_{hij},\sigma_{hij}^{2}),\;k=1,2,\ldots ,N_{hij},$$

where μ*_hij_* was a household mean of *X* and $\sigma_{hij}^{2}$ was a variance of *X* across individuals within households. These 2 parameters at the household level were given as

$$\mu_{hij}\sim N(\mu_{hi},\sigma_{hi}^{2}),\;\sigma_{hij}\sim N(10,0.2^{2}),$$

where μ*_hi_* and $\sigma_{hi}^{2}$ signify a mean and variance, respectively, of household means of *X* within clusters. The 2 parameters at the cluster level were generated as

$$\mu_{hi}\sim N(\mu_{h},\sigma_{h}^{2}),\;\sigma_{hi}\sim N(7,0.2^{2}),$$

where μ*_h_* and $\sigma_{h}^{2}$ denote a mean and variance, respectively, of cluster means of *X* within strata. These 2 parameters at the stratum level were specified as

$$\mu_{h}\sim N(130,2.5^{2}),\;\sigma_{h}\sim N(5,0.1^{2}).$$

All the specific numbers above were arbitrarily defined, except for the mean and standard deviation of μ*_h_* across strata, which reflect distributions of systolic blood pressures estimated from the National Health and Nutrition Surveys.^8^ As part of our attempt to investigate the performance of the sampling designs for categorical variables, the continuous *X* was further dichotomized to create a binary variable that indicated 1 for individuals having *X* equal to or greater than 140 and 0 for all other individuals.

### Sampling

A random sample of individuals was drawn from the population data, using the 2 sample designs mentioned above. Sampling was replicated 1000 times to obtain 1000 sample datasets for each sample design.

One of the sample designs followed that of the CSLC (Method 1): 100 clusters were selected from each of the 10 strata by systematic random sampling without replacement, and all households in the 1000 selected clusters were included in a sample. Sample weights for Method 1 were computed as the inverse of the proportion of the number of selected individuals to the population in each stratum. The weights were thus constant across observations within each stratum.

The other sample design was the 2-stage cluster sampling of households (Method 2), in which, after the data were sorted by identifiers of strata and clusters, clusters were selected throughout the 10 strata with probabilities proportional to the number of households without replacement in the first stage, and households were selected from each sample cluster by simple random sampling without replacement in the second stage. Five scenarios were established for Method 2 by using the total sample size of clusters in the first stage and a sampling fraction of households in the second stage: (1) 1000 clusters and 100%, (2) 2000 clusters and 50%, (3) 3000 clusters and 33%, (4) 4000 clusters and 25%, and (5) 5000 clusters and 20%. Sample weights for Method 2 were constructed as the inverse of the product of the probability of each cluster being selected and that of each household being sampled from each cluster. The weights were thus different across clusters, but were constant within clusters, for Method 2.

### Assessment

The mean of the continuous *X* and the proportion of its binary variable being equal to 1 (*X* ≥ 140) were estimated from each of the 1000 sample datasets to obtain a sampling distribution of 1000 estimates of each variable in total population and by strata. The survey commands of Stata were used to consider the complex survey designs including unequal probabilities of selection in the estimation procedure.^9^ All analyses were conducted with Stata/MP version 11.0 (StataCorp, College Station, TX, USA).

To compare the statistical performance of the 2 sample designs, the root mean squared errors (RMSEs) of the estimated means and proportions were computed from the sampling distributions. The RMSE is the square root of the sum of the variance and the squared bias of an estimator. In other words, it provides a summary measure of the overall accuracy of an estimator by integrating the standard deviation of a sampling distribution (efficiency) and the deviation of an expected value from a true value in the population (bias).^10^ In this study, the RMSE equals the variance because estimated means and proportions are unbiased under the simple weighted estimation for complex survey data.

## RESULTS

Table 1 shows the population size and basic statistics of *X* in the hypothetical population data. In total, the dataset had 34 262 865 individuals, 9 791 108 households, and 195 821 clusters. The population size by strata was comparable to the estimated Japanese population by prefecture in 2005: for instance, the smallest strata (ie, the fourth and ninth) were similar in size to Tottori and Shimane, whereas the 10th stratum was as large as Osaka prefecture excluding Osaka City.^3^ In the whole population, the mean of *X* was 129.8 (standard deviation, 13.4), and the proportion of *X* that was equal to or greater than 140 was 22%.

**Table 1.** Population size and basic statistics of a continuous variable *X* in a hypothetical population by strata

Stratum ID	Clusters	Households	Individuals	Mean of *X*	*X* ≥ 140 (%)
1	22 708	1 135 425	3 969 109	131.0	24.8
2	6043	302 308	1 058 277	126.0	14.4
3	31 176	1 558 708	5 455 087	128.3	18.9
4	4161	208 094	726 722	127.4	16.9
5	18 121	905 860	3 172 896	131.8	26.6
6	18 841	942 105	3 296 412	133.5	31.1
7	21 151	1 057 710	3 701 249	130.0	22.4
8	32 143	1 607 112	5 623 977	126.2	15.0
9	4538	226 915	794 826	129.1	20.4
10	36 939	1 846 871	6 464 310	131.6	26.2

Table 2 shows the average size of the 1000 sample datasets by strata and sample design. Method 1 sampled approximately 17 500 members of 5000 households in 100 clusters from each stratum. When Method 2 was used to sample 1000 clusters in total, the number of selected clusters was much lower than 100 in the smallest strata, while it increased in large strata by up to 89%.

**Table 2.** Average size of 1000 sample datasets by strata and sample design

Stratum ID	Method 1	Method 2 (by number of sample clusters)				

		1000	2000	3000	4000	5000
Clusters						
1	100	116	232	348	464	580
2	100	31	62	93	123	154
3	100	159	318	478	637	796
4	99	21	43	64	85	106
5	100	93	185	278	370	463
6	100	96	192	289	385	481
7	100	108	216	324	432	540
8	100	164	328	492	657	821
9	100	23	46	70	93	116
10	100	189	377	566	754	943
Total	999	1000	1999	3002	4000	5000
Households						
1	4994	5799	5830	5860	5958	5799
2	4999	1546	1554	1560	1587	1544
3	4995	7960	8003	8044	8179	7961
4	4937	1063	1070	1074	1092	1063
5	5002	4627	4651	4674	4752	4626
6	4993	4812	4838	4862	4944	4812
7	5001	5404	5432	5460	5552	5402
8	5001	8209	8253	8293	8431	8208
9	5001	1159	1165	1171	1191	1159
10	4986	9433	9483	9531	9691	9427
Total	49 909	50 012	50 279	50 529	51 377	50 001
Individuals						
1	17 434	20 272	20 377	20 490	20 825	20 269
2	17 409	5411	5439	5464	5559	5402
3	17 675	27 860	28 011	28 155	28 622	27 868
4	17 236	3712	3737	3750	3811	3714
5	17 417	16 207	16 288	16 372	16 639	16 200
6	17 496	16 833	16 928	17 018	17 300	16 837
7	17 556	18 906	19 002	19 109	19 429	18 901
8	17 672	28 727	28 883	29 019	29 508	28 732
9	17 383	4061	4081	4104	4171	4061
10	17 435	33 001	33 193	33 357	33 925	32 997
Total	174 713	174 990	175 939	176 838	179 789	174 981

Method 1, stratified sampling of a constant number of clusters; Method 2, two-stage cluster sampling of households.

Table 3 presents the RMSE of 1000 estimated means of *X* by strata and sample design. Using Method 2, sampling of 1000 clusters reduced the RMSE by 12% in the total population by changing the sampling method of clusters from simple random sampling of a fixed number of clusters in each stratum to sampling with probability proportional to size across strata. This sampling method also lowered the RMSE by 20% in large strata, although the RMSE considerably increased in small strata, mainly because of the abovementioned decrease in their sample size.

**Table 3.** Root mean squared error of 1000 estimates by strata and sample design

Stratum ID	Method 1	Method 2 (by number of sample clusters)				

		1000	2000	3000	4000	5000
Mean of continuous *X*						
1	0.522	0.504	0.320	0.282	0.237	0.232
2	0.498	0.931	0.708	0.593	0.542	0.541
3	0.540	0.418	0.297	0.272	0.224	0.186
4	0.653	1.067	0.756	0.684	0.546	0.557
5	0.502	0.569	0.438	0.375	0.330	0.282
6	0.486	0.534	0.400	0.322	0.301	0.276
7	0.511	0.459	0.342	0.285	0.250	0.214
8	0.526	0.406	0.327	0.258	0.221	0.204
9	0.554	1.050	0.830	0.679	0.556	0.563
10	0.475	0.374	0.264	0.246	0.202	0.172
Total	0.190	0.168	0.119	0.107	0.083	0.082
Proportion of *X* ≥ 140						
1	0.013	0.013	0.008	0.007	0.006	0.006
2	0.008	0.017	0.013	0.010	0.010	0.010
3	0.012	0.009	0.006	0.006	0.005	0.004
4	0.014	0.021	0.016	0.014	0.012	0.012
5	0.013	0.015	0.011	0.010	0.009	0.007
6	0.013	0.015	0.011	0.009	0.009	0.008
7	0.012	0.011	0.008	0.007	0.006	0.006
8	0.010	0.007	0.006	0.005	0.004	0.004
9	0.013	0.023	0.018	0.015	0.013	0.014
10	0.012	0.010	0.007	0.007	0.005	0.005
Total	0.004	0.004	0.003	0.003	0.002	0.002

Method 1, stratified sampling of a constant number of clusters; Method 2, two-stage cluster sampling of households.

As the number of sample clusters increased in Method 2, the RMSE of the estimated means of *X* for the total population continued to decline and stabilized at around two fifths of that of Method 1 when a quarter of households were sampled from 4000 clusters (Table 3). The RMSE of Method 2 also decreased across strata and was nearly equal to or less than that of Method 1 in all strata when 4000 clusters were selected in total. Similar results were obtained for the RMSE of the proportion estimates of *X* ≥ 140 both in the total population and by strata (Table 3).

## DISCUSSION

In designing national health surveys, it is essential to maximize the quality of health information, given the constraints on resources. This is particularly so for the CSLC because it is the largest health interview survey in Japan and serves as a sampling frame for some other national health surveys. The large-scale surveys of the CSLC currently employ an equal allocation of sample clusters to ensure equal errors of estimates across prefectures. The present simulation study confirmed that an alternative multistage probabilistic sampling might enhance the overall accuracy of estimates in a number of prefectures as well as in the whole population. A substantial part of this improvement was achieved by reducing variation in estimates by increasing the number of sample clusters and decreasing the sampling rate of households within clusters.

A major concern in introducing this alternative sample design is that traveling to more clusters might add to the burden on public health centers in large prefectures. However, this may not necessarily occur, because the sampling fraction of interview households decreases with the number of clusters selected. Moreover, it is not clear whether large prefectures currently share an appropriate burden for their population size or can still accept additional survey clusters to maintain balance with other prefectures.

Another concern regarding the implementation of the proposed survey design is that standard errors of estimates in small prefectures may become too large to be compared with those of other prefectures. However, our findings suggest that when the total number of clusters in a sample is adequate, the proposed sampling method also improves the variability of estimates in small prefectures. There is unlikely to be a large increase in the burden on small prefectures after switching to multistage proportional sampling, because the numbers of interview households and clusters do not exceed those of the conventional survey approach. Using the alternative survey design, a comparison of estimates at the subnational level may still be possible with reference to uncertainty intervals that appropriately reflect the population distribution and different sample sizes across prefectures. In addition, estimates for the total population that are derived without resorting to ratio estimates would theoretically have better comparability than those of small-scale surveys of the CSLC that employ a probabilistic sampling design. The introduction of this alternative method thus requires shifting the purpose of sampling designs from equal errors of estimates to the enhanced accuracy of parameter estimates across prefectures and in the whole population.

The Japanese health information system needs substantial reform in the design of national household surveys. To obtain nationally representative samples, a multistage probabilistic sampling survey design is becoming the norm for household health surveys across the world.^11^ It is also crucial to construct sample weights that account for any sampling errors and even to go as far as considering post-stratification weighting for nonresponse and noncoverage of subgroups.^12^ It is worthwhile to investigate how these elements of probabilistic sampling might be incorporated into the current sample design of the CSLC, so that information on population health could be generated with increased accuracy and compatibility while carefully considering resource implications.

The current study did have limitations that should be considered when interpreting the results. First, for ease of analysis, a continuous variable and its dichotomized variable were used for the assessment of sample designs. However, most of the variables collected by the large-scale CSLC were nominal or ordinal. It remains to be seen in future studies whether the findings from this study apply to multinomial and ordinal response scales. In addition, our estimates were based on simple weighted estimation techniques that took account of complex survey designs, although the large-scale CSLC employed ratio estimation using the number of household members as an auxiliary variable. Because ratio estimation is preferable only when variables of interest strongly correlate with the auxiliary variable,^13^ our estimation strategy is nevertheless appropriate for studying sample designs in the context of general variables that might be introduced in future health surveys. Second, this study did not incorporate post-stratification weights to adjust for bias caused by nonresponse. This is also a major issue in the redesign of the CSLC that will be examined in future studies. These limitations, however, are outweighed by the fact that this study is the first empirical assessment of sample designs used in Japanese health surveys. The simulation approach introduced in this article has proven to be a useful tool for testing the performance of designs of complex surveys and clinical trials.^7^ This analytic technique should be further applied in future research to investigate other important issues related to the sample design of the CSLC and other relevant surveys, such as how to ensure an adequate sample size for representing prefectures in smaller national surveys using the CSLC as a master sample.^1^

In conclusion, the alternative sampling approach proposed in this study was superior to the present CSLC strategy in obtaining accurate survey estimates of population parameters both by prefecture and in the entire population. Globally, multistage household surveys are now the standard and a key platform for understanding population health. Academics and policymakers should carefully examine the costs and benefits of this alternative survey strategy as they pertain to redesigning the CSLC to improve the quality of national health information and promote better understanding of population health in Japan.
